# Post‐PCI quantitative flow ratio predicts 3‐year outcome after rotational atherectomy in patients with heavily calcified lesions

**DOI:** 10.1002/clc.23816

**Published:** 2022-03-21

**Authors:** Wei You, Yuhe Zhou, Zhiming Wu, Peina Meng, Defeng Pan, Delu Yin, Song Yang, Xiangqi Wu, Fei Ye

**Affiliations:** ^1^ Division of Cardiology, Nanjing First Hospital Nanjing Medical University Nanjing China; ^2^ Division of Cardiology The First Affiliated Hospital of Anhui Medical University Hefei China; ^3^ Division of Cardiology The Affiliated Hospital of Xuzhou Medical University Xuzhou China; ^4^ Division of Cardiology The First People's Hospital of Lianyungang Lianyungang China; ^5^ Division of Cardiology Yixing People's Hospital Yixing China

**Keywords:** calcified lesion, patients, percutaneous coronary intervention, quantitative flow ratio, rotational atherectomy

## Abstract

**Background:**

The study sought to investigate the clinical predictive value of quantitative flow ratio (QFR) for the long‐term outcome in patients with heavily calcified lesions who underwent percutaneous coronary intervention (PCI) following rotational atherectomy (RA).

**Methods:**

In this retrospective study, 393 consecutive patients from 2009 to 2017 were enrolled. The QFR of the entire target vessel (QFRv) and the QFR of the stent plus 5 mm proximally and distally (in‐segment) (QFRi) were measured. The primary endpoint was target lesion failure (TLF), including target lesion‐cardiac death (TL‐CD), target lesion‐myocardial infarction (TL‐MI), and clinically driven‐target lesion revascularization (CD‐TLR).

**Results:**

A total of 224 patients with 224 calcified lesions completed the clinical follow‐up, and 52 patients had TLF. There was no significant difference in QFRv post‐PCI between non‐TLF and TLF groups (*p* > .05). However, QFRi post PCI was significantly higher in the non‐TLF group than in the TLF group. Multivariate Cox regression showed that QFRi post‐PCI was an excellent predictor of TLF after a 3‐year follow‐up (HR 1.7E^−8^ [5.3E^−11^–5.6E^‐6^]; *p* < .01). Furthermore, receiver‐operating characteristic curve analysis demonstrated that the optimal cutoff value of QFRi for predicting the long‐term TLF was 0.94 (area under the curve: 0.826, 95% confidence interval: 0.756–0.895; sensitivity: 89.5%, specificity: 69.2%; *p* < .01). The QFRi ≤ 0.94 post‐PCI was negatively associated with TLF, including TL‐CD, TL‐MI, and CD‐TLR (*p* < .01).

**Conclusions:**

QFRi post‐PCI showed a high predictive value for TLF for during a 3‐year follow‐up in patients who underwent PCI following RA; specifically, lower QFRi values post‐PCI were associated with worse TLF.

## 1 INTRODUCTION

Rotational atherectomy (RA) technique was first introduced more than 30 years ago and used to reduce plaque burden by the debulking idea before the stent era and by the modification idea in the current stage. However, both strategies failed to be proven superior to only balloon angioplasty, bare‐metal stent (BMS) implantation, or drug‐eluting stent (DES) implantation for coronary calcified lesions in a number of previous studies.^1^, ^2^, ^3^, ^4^, ^5^ At present, the significant indications of RA in daily practice are limited to heavily calcified lesions detected by coronary angiography (CAG) or intravascular imaging (IVI) as a bailout strategy.^6^ A previous study has shown that the final minimal luminal diameter (MLD) post‐BMS implantation following RA was the only significant independent predictor of event‐free survival.^7^ Even with DES used in the current stage for complex coronary lesions with/without the use of RA, nearly one‐third of patients experienced major adverse cardiac events (MACE, defined as the composite of death, myocardial infarction, and target vessel revascularization [TVR]) at 2‐year follow‐up.^8^ A question arises as to why percutaneous coronary intervention (PCI) of heavily calcified lesions can cause poor outcomes, we hypothesize that not only because of RA itself (which can cause thermal injury and additional vessel trauma and decrease the efficacy of DES in reducing neointimal growth) but unsatisfactory lesion preparation could also influence the final stent result. A physiological index such as fractional flow reserve (FFR) post‐PCI without RA has a good predictive value for the late outcome of target vessel failure (TVF, defined as the composite of cardiac death, target vessel‐related myocardial infarction, and clinically driven TVR).^9^ However, previous studies have not focused on the predictive value of physiological indexes post‐PCI on clinical outcome after the PCI procedure using RA due to complex manipulation of pressure wire.

Quantitative flow ratio (QFR), a novel physiological index derived from three‐dimensional (3‐D) angiographic analysis positively correlates with traditional invasive FFR, as shown in many studies.^10^, ^11^, ^12^, ^13^ The main advantage is that QFR measurement does not require hyperemia and a pressure wire. The online or offline analysis permits physiological guiding of PCI and retrospective physiological functional studies if the angiographic quality is satisfied.^10^, ^11^, ^12^, ^13^ Therefore, we designed a retrospective study to determine the predictive value of QFR post‐PCI for the target lesion failure (TLF) in the long‐term follow‐up. Currently, the QFR research has mainly focused on the vessel QFR, and there have been no studies on the stent QFR.^10^, ^11^, ^12^, ^13^, ^14^ Generally, the definition of the stent segment includes the stent segment and the 5‐mm area from its proximal and distal ends.^15^ In this study, we aimed to compare the predictive value of TLF between the vessel QFR and the stent QFR post‐PCI in patients with heavily calcified lesions using the RA technique.

## 2 MATERIALS AND METHODS

### 2.1 Study design

This was a retrospective cohort study. The study protocol was approved by the ethics committees of the four participating centers (Nanjing First Hospital, the Affiliated Hospital of Xuzhou Medical University, the First People's Hospital of Lianyungang, and Yixing People's Hospital), and the study was performed in accordance with the Declaration of Helsinki. All of the patients had signed informed consent twice during the in‐hospital period, that is, first, they signed informed consent for the RA procedure, and second, they signed informed consent for the clinical follow‐up in the retrospective study for the second time.

**Figure 1:**
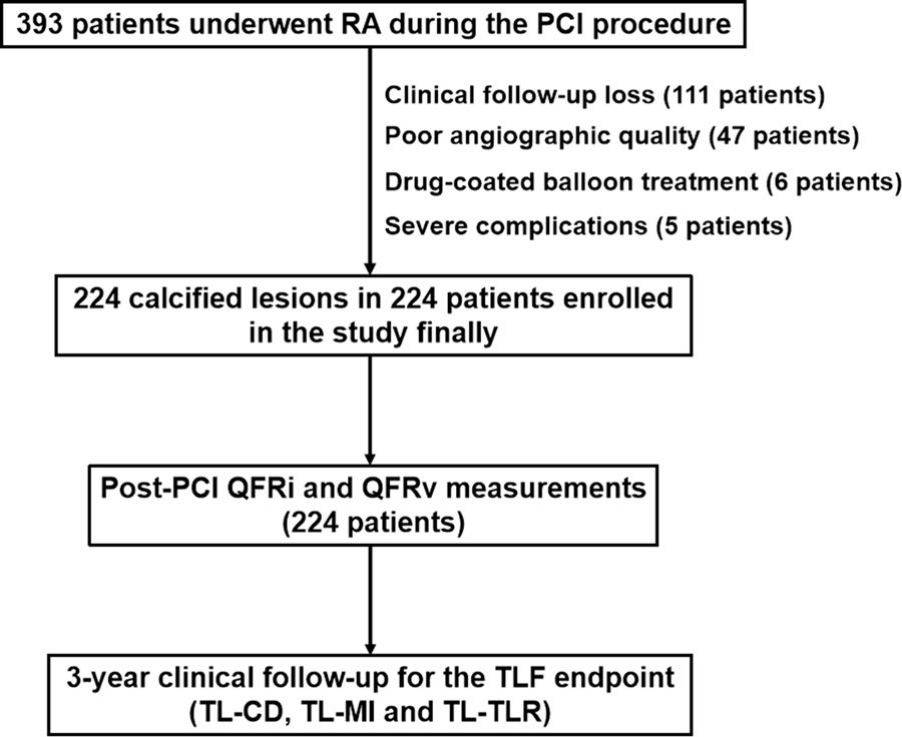
The study flowchart. PCI, percutaneous coronary intervention; QFRv, vessel quantitative flow ratio; QFRi, quantitative flow ratio in a segment; RA, rotational atherectomy; TLF, target lesion failure; TL‐CD, target lesion‐cardiac death; TL‐MI, target lesion‐myocardial infarction; TLR, target lesion revascularization

A total of 393 consecutive patients who had undergone RA management during the elective PCI for de novo calcified lesions in four hospitals from January 2009 to May 2017 were enrolled in this study. All clinical and PCI procedure variables were recorded from the follow‐up groups of the four hospitals (Nanjing First Hospital, the Affiliated Hospital of Xuzhou Medical University, the First People's Hospital of Lianyungang, and Yixing People's Hospital). Two experienced technicians blinded to follow‐up results independently measured clinical records and angiographic and QFR data. The study flowchart is shown in Figure 1.

### 2.2 Study population

We included patients treated with RA for target lesions with the indications of heavily calcified lesions (detected by angiography or IVI), or uncrossable or undilated calcified lesions. The exclusion criteria were as follows: severe complications of RA, such as perforation, post‐PCI slow flow or no flow (defined as coronary thrombolysis in myocardial infarction flow grade <3 or =0); culprit lesions that were not de novo lesions, such as in‐stent restenosis (ISR); PCI with drug‐coated balloon(s) or bioabsorbable scaffold implantation post‐RA; previous target vessel PCI; and a life expectancy < 12 months.

### 2.3 PCI procedure

All of the patients were administered 300 mg clopidogrel as a loading dose before CAG. Unfractionated heparin (100–120 μ/kg) was administered by bolus injection via sheath to maintain activated clotting time (ACT) > 300 s during the whole procedure. Standard selective CAG was performed via a radial approach with 6‐French catheters without a side hole in accordance with the routine practice. Intracoronary nitroglycerin (200 μg) was injected before selective CAG.

RA was performed using a Rotablator (Boston Scientific). The initial and final burr size with a floppy RotaWire (Boston Scientific) was at operator's discretion. The initial burr speed ranged from 135 000/min to 180 000/min, and the RA solution was a cocktail of verapamil, nitroglycerine, and heparin. Moreover, 98.7% of the procedures (221 of 224 patients) were performed with the radial approach, and in 94.2% of the patients (211 of 224 patients), guiding catheters were up to 6F in diameter. IVI was used for the PCI guidance in only 54.5% of the patients (122 of 224 patients). After the culprit vessel had been solved in 146 acute coronary syndrome (ACS) patients with multivessel disease at operator's discretion, the rota procedure in the nonculprit vessel was performed at the staged PCI. All the lesions were implanted with the new‐generation DES, and high‐pressure post‐dilatation with a noncompliant balloon was applied completely.

### 2.4 Intravascular ultrasound (IVUS) procedure

After intracoronary injection of nitroglycerin (100–200 mg), an IVUS catheter was pushed at least 10 mm distal to the lesion or stent edge. IVUS images were obtained through the automatic pullback (0.5 mm/s) by a commercially available imaging system with a 40‐MHz mechanical transducer (Boston Scientific) for measuring on‐site. All of the IVUS images were stored on a DVD for offline measurements. Minimal stent diameter, maximal stent diameter, and minimal stent area were measured, and the stent eccentricity index was calculated as minimal stent diameter/maximal stent diameter.

### 2.5 Quantitative coronary angiography (QCA) and QFR measurements

Two high‐quality coronary angiographic projections at least 25° apart post‐PCI are required to satisfy the requirements of QCA and QFR analyses, which were performed offline by two experienced technicians (with a high interrater agreement in all cases [*к* > 0.90]) at Nanjing Heart Center (core lab) using AngioPlus software (Pulse Medical Imaging Technology) as previously described.^10^, ^14^ QCA data included pre‐ and post‐PCI proximal and distal reference vessel diameter (RVD), total stent length (TSL), and in‐stent MLD immediately post‐PCI. In the present study, we considered traditional QFR of the entire target vessel starting from the most proximal available segment until the distal part where the vessel diameter was ≥ 1.5 mm (vessel QFR [QFRv]), and the QFR in a segment was measured from 5 mm proximal to 5 mm distal to the stent edge (stent QFR [QFRi]) for subsequent analysis.^11^, ^14^, ^15^ Figure S1 shows how QFRv and QFRi post‐PCI were measured on a representative case. The figure was generated by AngioPlus QFR 1.0 software (Pulse Medical Imaging Technology, Shanghai Co., Ltd.) as previously described.^14^

### 2.6 Clinical data collection and study endpoints

Patients' clinical, demographic data, cardiovascular risk factors, medical treatment, and clinical follow‐up data were collected from the follow‐up group in the above four hospitals. Clinical follow‐up was performed for all 224 patients, and CAG follow‐up was conducted in 112 patients. The CAG procedure was performed 1 year after PCI. The median clinical follow‐up duration was 1095 days (interquartile range [IQR]: 1095–1095 days).

The primary endpoint was TLF at the 3‐year clinical follow‐up. TLF was defined as a composite of target lesion‐related cardiac death (TL‐CD), target lesion‐related myocardial infarction (TL‐MI), and clinically driven‐target lesion revascularization (CD‐TLR) by coronary artery bypass graft surgery (CABG) or PCI. TL‐CD was defined as all‐cause death unless an unequivocal noncardiac cause or nontarget lesion‐related cause was established. The definition of MI was in accordance with the guidelines of the European Society of Cardiology (ESC).^16^ TL‐MI occurrence without an identified culprit vessel was considered as a target lesion‐related. CD‐TLR was defined as any repeated revascularization in the presence of a lesion with percentage diameter stenosis (%DS) > 90%, or %DS > 50% accompanied with relevant evidence of angina plus objective signs of ischemia at rest or during exercise, or relevant positive ischemic evidence on any noninvasive functional stress test.^17^, ^18^ All of the events were judged by an independent clinical event committee that was blinded to the PCI procedure and QFR and QCA data.

### 2.7 Statistical analysis

According to the ROTAXUS trial,^8^ the MACE rate was 29.4% in patients with heavily calcified lesions after the RA procedure at a 2‐year follow‐up. The predictor had a standard deviation of 0.05, and the hazard ratio (HR) was set at 1.0. We tested the hypothesis using a 5% significance level with a two‐sided Wald test. As a result, the sample size of 224 was calculated with 1.00 power by PASS11.0 software (NCSS, LLC).

Categorical variables were expressed as counts with percentages, whereas continuous variables were expressed as mean with standard deviation (SD) or median with IQRs. Categorical variables were compared using the *χ* ^2^ test. The Kolmogorov–Smirnov test was used to assess the distributions of continuous variables. Continuous variables were expressed as mean ± SD for normally distributed data and were compared using the Student's *t* test. Data that were not normally distributed were expressed as medians and were compared using the Mann–Whitney *U* test. The Kaplan–Meier method was used to derive the event rates at the follow‐up and to plot time‐to‐event curves, which were then compared by the log‐rank test. To study TLF predictors, a univariate Cox regression was performed. Variables that were found to be significant were entered into a multivariate model. Their outputs included HR, 95% confidence interval (CI), and *p* value. The receiver operating characteristic curve was used to compare the variables' predictive ability of the rates of TLF. All of the statistical tests were two‐tailed. Statistical significance was set at .05. For the statistical analysis, the SPSS version 24.0 (SPSS Institute Inc.) was used.

## 3 RESULTS

### 3.1 Basic clinic, procedural, and QCA data of patients with heavily calcified lesions with and without TLF after RA

A total of 71.8% of the patients (282/393) finished clinical follow‐up at 3 years if they had no symptoms, or at less than 3 years if they felt chest pain during exercise or had other clinical ischemic evidence or acute coronary syndrome (ACS). Among them, 47 cases had a low‐quality CAG, which did not satisfy the criterion for QFR measurement; six patients were treated with a drug‐coated balloon without DES implantation; two patients had perforation as a complication; and three patients had severely slow or no flow post‐RA combined with periprocedural MI. Finally, 224 patients were eligible for inclusion in the current study. Among 172 patients with multivessel disease, 170 underwent complete revascularization.

No significant differences between TLF and non‐TLF groups were detected in age, gender, family history of coronary artery disease (CAD), cardiovascular risk factors (hyperlipidemia, hypertension, diabetes, current smoker, renal insufficiency, and hemodialysis), clinical diagnosis (stable angina pectoris [SAP], unstable angina pectoris [UAP], non‐ST‐segment elevation myocardial infarction [NSTEMI], and ST‐segment elevation myocardial infarction [STEMI]), antiplatelet therapy, statin therapy, single‐vessel disease, multivessel disease, pre‐PCI proximal and distal RVDs, pre‐PCI DS, lesion length, pre‐dilated balloon diameter, pre‐dilated pressure, post‐dilated balloon diameter, post‐PCI proximal and distal RVDs, total stent length, average stent diameter, stent number, QFRv post‐PCI, burr to vessel diameter ratio, imaging use, cutting balloon (CB) use, and blood flow velocity (FV) post‐PCI (*p* > .05) (Table 1). However, the target vessel location in the TLF group was significantly different from that in the non‐TLF group (*p* < .05). Post‐dilated pressure and post‐PCI DS of the TLF group were notably higher than those in the non‐TLF group, and post‐PCI MLD and QFRi post‐PCI of the TLF group were markedly lower (*p* < .05 or *p* < .01) (Table 1).

**Table 1:** Basic clinic, procedural, and QCA data of patients with heavily calcified lesions with and without TLF after RA

Variable	TLF (*N* = 52)	Non‐TLF (*N* = 172)	*p* value
Age, years	71.5 ± 8.0	71.0 ± 7.6	.690
Male (%)	33 (63.5%)	119 (69.2%)	.439
Family history of CAD (%)	2 (3.8%)	5 (2.9%)	.665
CV risk factors			
Hyperlipidemia (%)	38 (73.1%)	117 (68.0%)	.489
Hypertension (%)	36 (69.2%)	132 (76.7%)	.273
Diabetes (%)	23 (44.2%)	68 (39.5%)	.546
Current smoker (%)	21 (40.4%)	70 (40.7%)	.968
Renal insufficiency (%)	5 (9.6%)	9 (5.2%)	.323
Hemodialysis (%)	2 (3.8%)	4 (2.3%)	.625
Clinical diagnosis			.512
SAP (%)	11 (21.2%)	29 (16.9%)	
UAP (%)	27 (51.9%)	105 (61.0%)	
NSTEMI (%)	5 (9.6%)	19 (11.0%)	
STEMI (%)	9 (17.3%)	19 (11.0%)	
Antiplatelet therapy			
Aspirin (%)	52 (100%)	172 (100%)	–
Clopidogrel/Ticagrelor (%)	52 (100%)	172 (100%)	–
Clopidogrel (%)	32 (61.5%)	113 (65.7%)	.582
Statin therapy (%)			.128
Atorvastatin	30 (57.7%)	88 (51.2%)	
Rosuvastatin	20 (38.5%)	77 (44.8%)	
Simvastatin	0 (0%)	6 (3.5%)	
QCA data			
Vessel disease number			.250
Single vessel disease (%)	9 (17.3%)	43 (25.0%)	
Multiple vessel disease (%)	43 (82.7%)	129 (75.0%)	
Target vessels			.020
LAD (%)	34 (65.4%)	143 (83.1%)	
RCA (%)	14 (26.9%)	21 (12.2%)	
LCX (%)	4 (7.7%)	8 (4.7%)	
Pre‐PCI distal RVD (mm)	2.4 (2.1,2.6)	2.3 (2.0,2.7)	.228
Pre‐PCI proximal RVD (mm)	2.8 (2.3,3.2)	2.8 (2.4,3.2)	.465
Pre‐PCI MLD (mm)	0.6 (0.5,0.8)	0.5 (0.6,0.7)	.055
Pre‐PCI DS (%)	74.7 (66.7,79.4)	75.9 (70.0,80.8)	.250
Lesion length (mm)	56.2 (45.8,69.8)	56.2 (43.3,70.0)	.734
Procedure data			
Initial burr size (mm)	1.40 ± 0.20	1.42 ± 0.18	.474
Final burr size (mm)	1.48 ± 0.22	1.50 ± 0.18	.516
Pre‐dilated balloon diameter (mm)	2.63 ± 0.44	2.66 ± 0.38	.549
Pre‐dilated pressure (atm)	19.65 ± 2.95	18.91 ± 2.63	.084
Post‐dilated balloon diameter (mm)	3.47 ± 0.63	3.49 ± 0.58	.827
Post‐dilated pressure (atm)	20.65 ± 1.91	19.95 ± 1.71	.013
Post‐PCI distal RVD (mm)	2.6 (2.2,3.0)	2.6 (2.3,3.0)	.787
Post‐PCI proximal RVD (mm)	3.1 (2.7,3.7)	3.1 (2.6,3.5)	.218
Post‐PCI MLD (mm)	1.6 (1.4,2.0)	1.9 (1.7,2.2)	.001
Post‐PCI DS (%)	32.1 (26.9,39.5)	26.4 (22.6,30.8)	≤.001
Total stent length (mm)	63.5 (50.5,76.5)	61.5 (47.8,76.0)	.461
Average stent diameter (mm)	3.0 (2.8,3.3)	2.9 (2.7,3.1)	.259
Stent number	2.0 (2.0,3.0)	2.0 (2.0,3.0)	.326
QFRv post PCI	0.848 ± 0.144	0.904 ± 0.100	.079
QFRi post PCI	0.915 ± 0.066	0.978 ± 0.034	≤.001
Burr to vessel ratio	0.63 (0.52,0.70)	0.65 (0.56,0.74)	.156
Imaging use (%)	30 (57.7%)	92 (53.5%)	.636
CB use (%)	8 (15.4%)	24 (14.0%)	.822
FV post PCI (m/s)	0.21 (0.16,0.23)	0.18 (0.14, 0.23)	.101

*Note*: Data were expressed as *n* (%), mean ± SD, and median (quartile 1, quartile 3).

Abbreviations: CB, cutting balloon; CV, cardiovascular; DS, diameter stenosis; FV, flow velocity; LAD, left anterior descending coronary artery; LCX, left circumflex coronary; MLD, minimal luminal diameter; NSTEMI, non‐ST‐segment elevation myocardial infarction; PCI, percutaneous coronary intervention; QCA, quantitative coronary angiography; QFRi, quantitative flow ratio in a segment; QFRv, vessel quantitative flow ratio; RCA, right coronary artery; RVD, reference vessel diameter; SAP, stable angina pectoris; *SD*, standard deviation; STEMI, ST‐segment elevation myocardial infarction; TLF, target lesion failure; UAP, unstable angina pectoris.

Furthermore, we analyzed the minimal stent diameter, maximal stent diameter, minimal stent area, and stent eccentricity index in patients with heavily calcified lesions immediately after PCI. The minimal stent diameter, minimal stent area, and stent eccentricity index in the non‐TLF group were larger than those in the TLF group (*p* < .05). However, there was no significant difference in maximal stent diameter between the two groups (*p* > .05) (Table S1). At 1‐year follow‐up, there were no differences in proximal RVD and distal RVD between the TLF group and the non‐TLF group (*p* > .05). However, MLD in the TLF group was smaller than that in the non‐TLF group, and DS in the TLF group was larger (*p* < .05) (Table S2).

These results indicated that TLF occurrence was closely related to post‐dilated pressure, MLD, DS, and QFRi post‐PCI, and MLD and DS 1 year after PCI in patients with heavily calcified lesions after RA. Moreover, lower minimal lumen diameter, minimal stent area, and eccentricity index were associated with a failure rate of the target lesion.

### 3.2 Cox regression analysis of factors associated with TLF and their predictive value analyzed by ROC curve in patients with heavily calcified lesions after RA at 3‐year follow‐up

**Figure 2:**
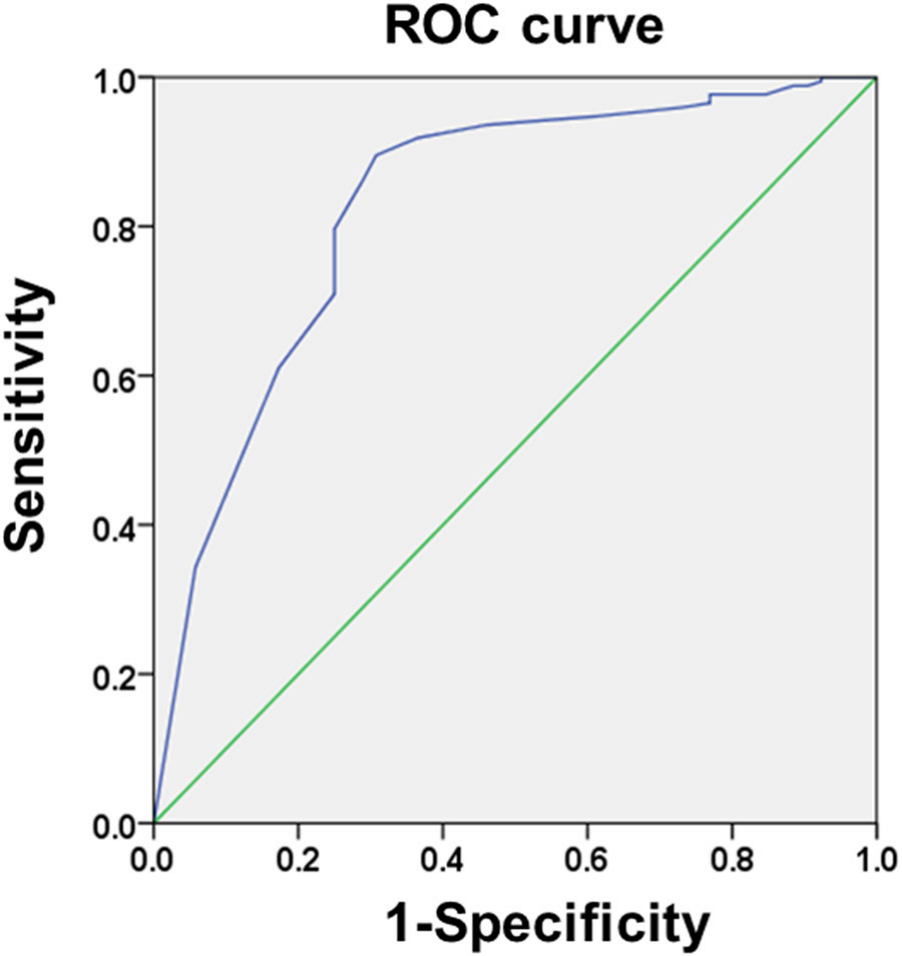
ROC curve analysis of QFRi post‐PCI to predict TLF in patients with heavily calcified lesions after RA at the 3‐year follow‐up. The cutoff value of QFRi post‐PCI for predicting TLF was 0.94 (sensitivity: 0.895, specificity: 0.692). PCI, percutaneous coronary intervention; QFRi, quantitative flow ratio in a segment; RA, rota atherectomy; ROC, receiver operating characteristic curve; TLF, target lesion failure

To further analyze factors associated with TLF in patients with heavily calcified lesions after RA at 3‐year follow‐up, we used the univariate and multivariate Cox regression methods. As shown by the univariate Cox regression analysis, post‐dilated pressure, MLD, DS, QFRv, and QFRi post‐PCI, as well target vessel LAD, were able to predict TLF in these patients after RA (*p* < .05 or *p* < .01) (Table 2). Next, these six factors were used in the multivariate Cox regression analysis. DS and QFRi post‐PCI, and target vessel LAD were better predictors of TLF than the other three factors (postdilated pressure, and MLD and QFRv post‐PCI) in these patients (*p* < .01) (Table 2). The ROC analysis showed that the cutoff value of QFRi post‐PCI was 0.94, with a sensitivity of 89.50%, specificity of 69.20%, Youden index of 0.587, and area under the curve (AUC) of 0.826 (95% CI: 0.756–0.895) for predicting TLF at the 3‐year follow‐up (*p* < .01) (Figure 2).

**Table 2:** Predictors of TLF were analyzed by the Cox regression method

Variables	Univariate analysis	*p* Value	Multivariate analyses	*p* Value
	HR (95% CI)		HR (95% CI)	
Male (%)	1.246 (0.708–2.191)	.445		
Age (years)	1.007 (0.972–1.044)	.696		
Hypertension (%)	0.738 (0.409–1.329)	.311		
Hyperlipidemia (%)	1.236 (0.670–2.282)	.498		
Diabetes (%)	1.184 (0.685–2.046)	.546		
Smoking (%)	0.984 (0.566–1.713)	.956		
Single‐vessel lesion (%)	0.643 (0.313–1.318)	.228		
Multiple‐vessel lesion (%)	1.556 (0.758–3.192)	.228		
Target‐lesion length (mm)	1.001 (0.989–1.013)	.915		
pre‐PCI DS (%)	0.989 (0.961–1.018)	.464		
Initial burr size (mm)	0.539 (0.112–2.600)	.441		
Final burr size (mm)	0.548 (0.120–2.497)	.437		
Pre‐dilated balloon diameter (mm)	0.779 (0.384–1.581)	.490		
Pre‐dilated pressure (atm)	1.089 (0.992–1.196)	.073		
Post‐dilated balloon diameter (mm)	0.922 (0.575–1.478)	.735		
Post‐dilated pressure (atm)	1.188 (1.038–1.360)	.012	1.081 (0.949–1.231)	.241
Target‐vessel stent length (mm)	1.003 (0.991–1.015)	.608		
MLD post PCI (mm)	0.239 (0.110–0.518)	≤.001	1.330 (0.572–3.094)	.508
DS post PCI (%)	1.090 (1.060–1.121)	≤.001	1.067 (1.036–1.100)	≤.001
QFRv post PCI	0.048 (0.008–0.290)	.001	2.382 (0.145–39.073)	.543
QFRi post PCI	1.2E^−07^ (3.0E^−09^–4.8E^−06^)	≤.001	1.7E^−8^ (5.3E^−11^–5.6E^−6^)	≤.001
B to V ratio	0.152 (0.020–1.138)	.067		
Imaging use (%)	1.156 (0.667–2.004)	.605		
CB use (%)	1.068 (0.503–2.269)	.864		
LAD (%)	0.439 (0.248–0.778)	.005	0.383 (0.210–0.697)	.002
FV post PCI (m/s)	15.642 (0.563–434.677)	.105		

Abbreviations: B to V, burr to vessel; CB, cutting balloon; CI, confidential interval; DS, diameter stenosis; FV, flow velocity; HR, hazard ratio; LAD, left anterior descending coronary artery; MLD, minimal luminal diameter; PCI, percutaneous coronary intervention; QFRi, quantitative flow ratio in a segment; QFRv, vessel quantitative flow ratio; TLF, target lesion failure.

These results suggested that QFRi post‐PCI was an excellent predictor of TLF in patients with heavily calcified lesions after RA at the 3‐year follow‐up.

### 3.3 Clinical outcome in patients with heavily calcified lesions after RA at the 3‐year follow‐up

According to the QFRi post‐PCI cutoff value of 0.94, we divided these patients into high‐ and low‐QFR groups. The incidence rate of TLF, TL‐CD, TL‐MI, and TLR in the high‐QFR group were significantly lower than those in the low‐QFR group (*p* < .05 or *p* < .01) (Table 3).

**Table 3:** The clinical outcome between the high‐ and low‐QFR groups after about 3 years post‐PCI

	QFR > 0.94 (*N* = 170)	QFR ≤ 0.94 (*N* = 54)	*p* value
TLF (%)	16 (9.4%)	36 (66.7%)	≤.001
TL‐CD (%)	6 (3.5%)	6 (11.1%)	.042
TL‐MI (%)	5 (2.9%)	11 (20.4%)	≤.001
TLR (%)	10 (5.9%)	30 (55.6%)	≤.001

*Note*: Data were expressed as *n* (%).

Abbreviations: PCI, percutaneous coronary intervention; QFRi, quantitative flow ratio; TL‐CD, target lesion‐cardiac death; TL‐MI, target lesion‐myocardial infarction; TLF, target lesion failure; TLR, target lesion revascularization.

**Figure 3:**
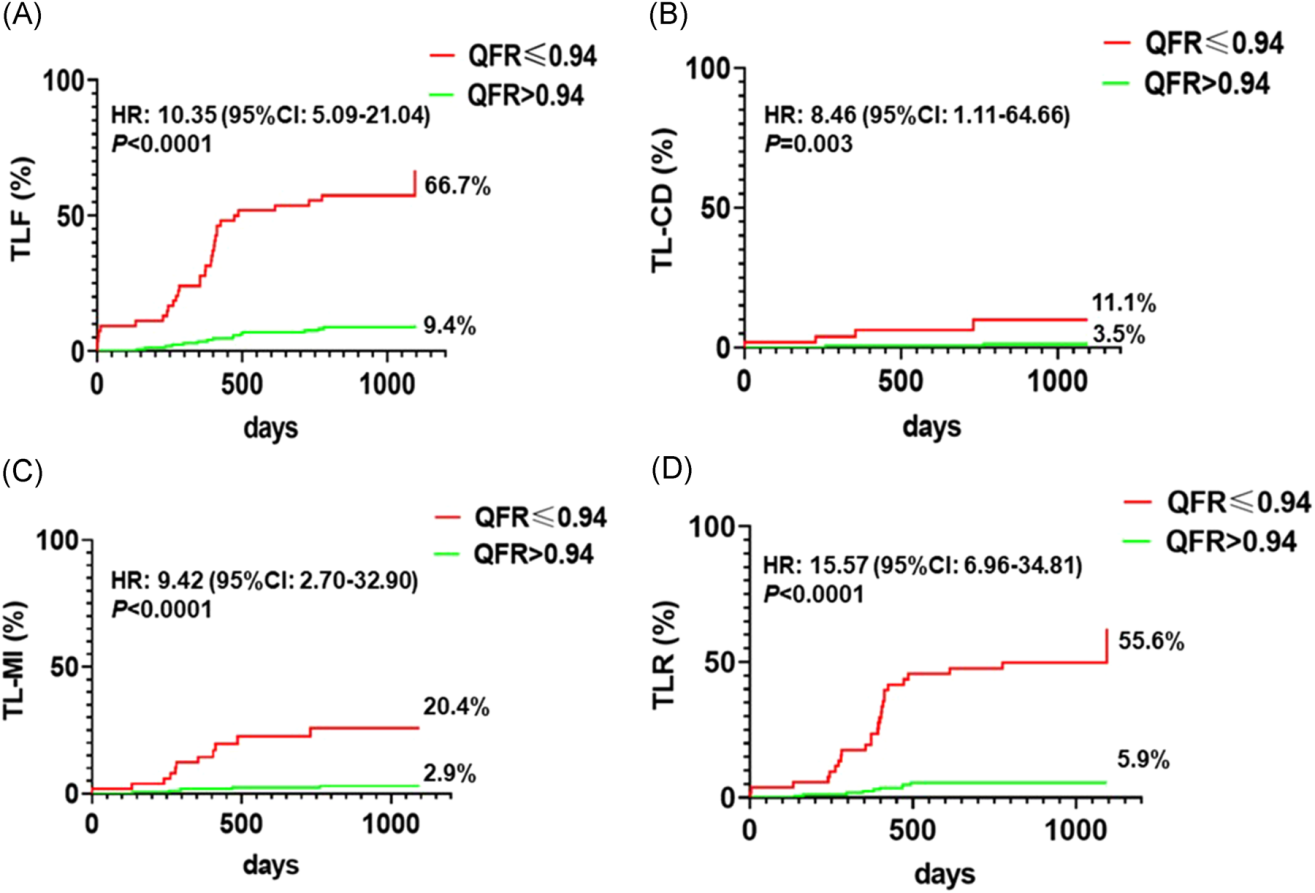
TLF and its compositions were analyzed by the Kaplan–Meier curves between the high‐ and low‐QFRi groups at the 3‐year follow‐up. (A) TLF; (B) TL‐CD; (C) TL‐MI; (D) TLR. TLF, target lesion failure; TL‐CD, target lesion‐cardiac death; TL‐MI, target lesion‐myocardial infarction; TLR, target lesion revascularization

TLF and its compositions analyzed using the Kaplan–Meier curves are shown in Figure 3. The TLF ratio in the low‐QFRi group was significantly higher than in the high‐QFRi group (66.7% vs. 9.4%, *p* < .0001, HR: 10.35 [95% CI: 5.09–21.04]). Further analysis showed that TL‐CD (11.1% vs. 3.5%, *p* = .003, HR: 8.46 [95% CI: 1.11–64.66]), TL‐MI (20.4% vs. 2.9%, *p* < .0001, HR: 9.42 [95% CI: 2.70–32.90]), and TL‐TLR (55.6% vs. 5.9%, *p* < .0001, HR: 15.57 [95% CI: 6.96–34.81]) were higher in the low‐QFRi group than in the high‐QFRi group.

These results revealed that the cutoff value (0.94) after QFRi post PCI could differentiate the risk level of TLF occurrence in patients with heavily calcified lesions after RA at the 3‐year follow‐up.

## 4 DISCUSSION

For the first time, we investigated the low QFRi post‐PCI ( ≤ 0.94) in patients who underwent RA and the second‐generation DES implantation showed a substantial predictive value of high TLF at the 3‐year follow‐up. The analysis of QFR computation post‐PCI for evaluating lesion burden of the residual physiological vascular is more convenient than the traditional FFR measurement. Previous studies have shown a significant association between the low FFR measurement post‐PCI without RA and the high risk of clinical adverse events at mid‐ and long‐term follow‐up.^19^, ^20^, ^21^, ^22^ Due to different sensitive points of pressure wire location in the target vessel, FFR measurement of the target vessel would be influenced not only by the stent segment and the non‐stent segment but also by the distal vessel disease and microcirculatory dysfunction. Therefore, a wide range of cutoff values of FFR post‐PCI were described in different studies. The physiological function test of a moderate coronary lesion is a significant indication for PCI guidance in the current clinical practice.^23^ FFR has shown an outstanding value for predicting the late outcome post‐PCI in a number of previous studies not only for single‐vessel disease but also for multivessel disease. Considering the complex manipulation of FFR measurement by the pressure wire, the utilization rate is currently very low. A retrospective study of the physiological function is impossible if FFR measurement was not performed during the previous procedure; in contrast, it can still be done with the QFR appearance if the previous CAG quality meets the measuring requirements.

Compared with non‐ or mildly calcified lesions, moderately or severely calcified lesions with/without RA led to poor clinical outcomes in previous studies, even though the second‐generation DES was used. This is because the lesion was poorly prepared, causing the stent underexpansion and malposition. RA may improve these scenarios, but it still fails to correlate with optimized clinical outcomes. In the present study, we measured two kinds of QFR (QFRv and QFRi) to differentiate the stenting effect and residual lesion burden; we found a high correlation between low post‐PCI QFRi and high TLF at the 3‐year follow‐up for patients who underwent RA, which is consistent with previous studies without RA.^13^, ^19^, ^20^, ^21^, ^22^ The optimal cutoff value of QFRi post‐PCI was 0.94 based on the ROC curve analysis, which is slightly high compared with the findings of the previous studies without RA (QFR: 0.85–0.92).^13^ The difference was likely caused by the inconsistency in observational indicators and clinical endpoints among different studies. Previous studies have mainly focused on the correlation between FFR/QFRv and MACE, while we primarily observed the correlation between QFRi and TLF. Patients with heavily calcified lesions after RA were more likely to have insufficient stent expansion and apposition than patients with non‐ or mildly calcified lesions. Such a suboptimal stent deployment would cause increased stent‐related events. Therefore, the stent‐related physiological index (QFRi) might predict late TLF more accurately than QFRv post‐PCI.

LAD as the RA's target vessel showed significantly low TLF compared with left circumflex artery (LCX) and right coronary artery (RCA) in our observational study. The reason might be that the sample size was small, and that the LAD's RA was used more often in the non‐TLF group than in the TLF group (83.1% vs. 65.4%). Low burr‐to‐vessel ratio causes a bad plaque modification, which leads to insufficient lesion preparation and stent underexpansion and malposition.^4^, ^24^ High DS post‐PCI is a predictor of in‐stent restenosis and stent thrombosis.^25^ Therefore, in the present study, DS post PCI in the TLF group was notably high compared with that in the non‐TLF group, and the burr‐to‐vessel ratio was likely to decrease in the TLF group. Additionally, the postdilated pressure of the balloon in the TLF group was higher than that in the non‐TLF group, suggesting that the lesion preparation was insufficient in the TLF group. Still, DS post‐PCI and postdilated pressure were both able to predict TLF in patients with heavily calcified lesions after RA at the 3‐year follow‐up by the univariate Cox regression analysis.

With the progress of interventional treatment of heavily calcified lesions, once the post‐PCI QFR value is found to be less than the cutoff value from the present study, it is suggested to further use effective therapeutic methods, such as shockwave balloon, to improve the final post‐PCI QFR value and achieve the optimization of therapy. Additionally, QFR measurement was very effective and simple in predicting future clinical prognosis. Thus, it is recommended to provide routine post‐PCI QFRi guidance for patients with complex PCI (such as RA) in daily practice. Based on the new concept suggested in the present study, the prospective randomized clinical trials should be designed in the future to treat patients with heavily calcified lesions using RA under the guidance of post‐PCI QFRi.

### 4.1 Limitations

This study had some limitations. First, our study was a retrospective study in which the imaging use ratio was about 50%, and <60% of the cases had clinical follow‐up, and 50% of the cases had the angiographic follow‐up. The reasons for this situation were the increased financial burden and unconventional clinical and CAG follow‐up. Second, this study did not provide post‐PCI FFR values. Finally, there was no established methodology for determining the QFR values of the stented segment.

## 5 CONCLUSIONS

QFRi post‐PCI showed a high predictive value for the long‐term clinical outcome in patients who underwent RA during the PCI procedure. Besides, the lower QFRi post‐PCI was associated with higher TLF. QFRi could be applied for evaluating the coronary stenting outcome in patients who underwent RA during the complex PCI.

## CONFLICT OF INTERESTS

The authors declare no conflict of interest.

## Supporting information

Supporting information.

## Data Availability

The data in support of the results are available from the corresponding author on reasonable request.
