# Supplementary material for: Post‐PCI quantitative flow ratio predicts 3‐year outcome after rotational atherectomy in patients with heavily calcified lesions
Source: Clin Cardiol. 2022 Mar 21;45(5):558–66. doi: 10.1002/clc.23816 (PMC9045081; doi:10.1002/clc.23816)
Supplement: Supplementary file 1 — Supporting information. [file CLC-45-558-s001.docx]

**SUPPLEMENTAL TABLE 1 Condition of minimum stent area immediately post-PCI between the TLF and non-TLF groups after the IVUS use in patients with heavily calcified lesions.**

| IVUS use | TLF group  (N=30) | Non-TLF group  (N=92) | *p* value |
| --- | --- | --- | --- |
| Minimal stent diameter (mm) | 1.98 (1.61, 2.32) | 2.23 (1.94, 2.50) | 0.024 |
| Maximal stent diameter (mm) | 2.45 (2.01, 2.98) | 2.55 (2.32, 2.96) | 0.112 |
| Minimal stent area (mm) | 3.42 (2.62, 4.93) | 4.30 (3.60, 5.63) | 0.010 |
| Eccentricity index | 0.79 (0.73, 0.85) | 0.85 (0.78, 0.90) | 0.047 |

Data were expressed as median (quartile 1, quartile 3). Abbreviations: PCI, percutaneous coronary intervention; TLF, target lesion failure; IVUS: intravascular ultrasound.

**SUPPLEMENTAL TABLE 2 QCA data after 1-year follow-up between patients with and without TLF.**

| Variable | TLF (N=26) | Non-TLF (N=86) | *p* value |
| --- | --- | --- | --- |
| Distal RVD (mm) | 2.5 (2.2, 3.0) | 2.5 (2.3, 3.0) | 0.740 |
| Proximal RVD (mm) | 3.1 (2.7, 3.5) | 3.1 (2.7, 3.4) | 0.501 |
| MLD (mm) | 1.2 (1.0, 1.3) | 1.8 (1.5, 2.1) | 0.000 |
| DS (%) | 56.0 (48.5, 62.8) | 29.2 (23.1, 34.8) | 0.000 |

Data were expressed as median (quartile 1, quartile 3). Abbreviations: DS, diameter stenosis; MLD, minimal luminal diameter; QCA, quantitative coronary angiography; RVD, reference vessel diameter; TLF, target lesion failure; UAP, unstable angina pectoris.


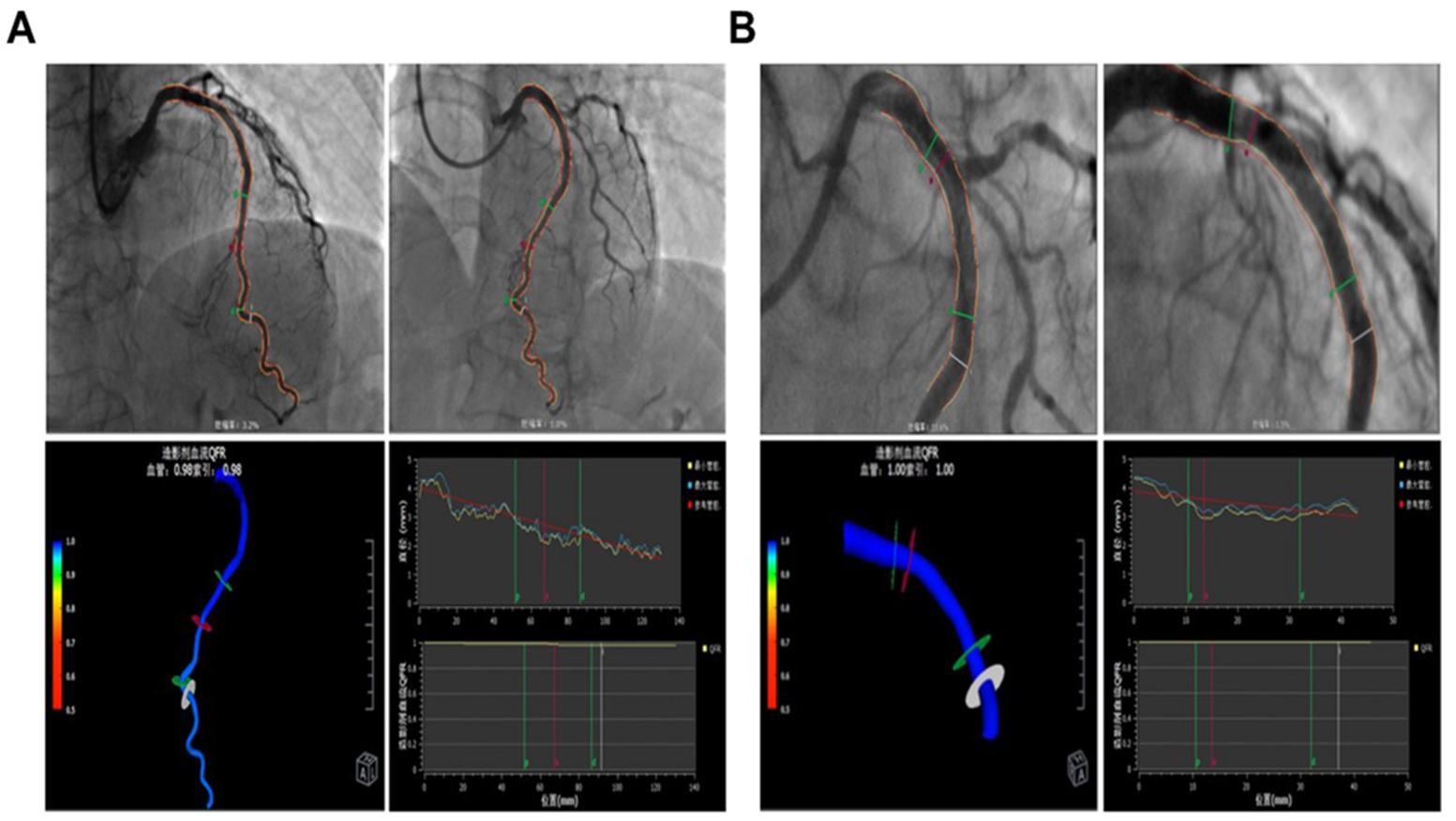


**SUPPLEMENTAL FIGURE 1 A representative case showed how QFRv and QFRi were measured post-PCI.** A. The angiographic images in both left (LAO 20° + cranial 30°) and right (RAO 20° + cranial 30°) cephalic positions of a 63-year-old male patient were used to analyze QFRv by the AngioPlus QFR 1.0 software, and the value of QFRv was 0.98; B. The above-mentioned angiographic images of the patient were used to analyze QFRi by the QFR software, and the value of QFRi was 1.00. LAO, left anterior oblique; QFRv, vessel quantitative flow ratio; QFRi, quantitative flow ratio in a segment; RAO, right anterior oblique.
